# AI-Driven Bone and Marrow Segmentation on FLT-PET/CT: Technical Multi-organ Validation in AML and HCT

**DOI:** 10.21203/rs.3.rs-9077609/v1

**Published:** 2026-04-19

**Authors:** Malakeh Malekzadeh, Hemendra Ghimire, Karteek Popuri, Kazuki Fujita, Amandeep Salhotra, Dave Yamauchi, Bihong Chen, Jerry Froelich, Guy Storme, Anthony Stein, Mirza Faisal Beg, Jeffrey Wong, Monzr M Al Malki, Susanta K Hui

**Affiliations:** City of Hope National Medical Center; City of Hope National Medical Center; Memorial University: Memorial University of Newfoundland; City of Hope National Medical Center; City of Hope National Medical Center; City of Hope National Medical Center; City of Hope National Medical Center; University of Minnesota; Universitair Ziekenhuis Brussel; City of Hope National Medical Center; Simon Fraser University; City of Hope National Medical Center; City of Hope National Medical Center; City of Hope National Medical Center

**Keywords:** [18F] 3’-deoxy-3’-fluorothymidine, PET/CT, Multiple Organ Segmentation, Bone Marrow Examination

## Abstract

**Background:**

[18F] 3’-deoxy-3’-fluorothymidine positron emission tomography (FLT-PET) is valuable for detecting acute myeloid leukemia (AML) and monitoring stem cell engraftment after hematopoietic stem cell transplant (HCT) by assessing cellular proliferation in marrow-rich tissues. Reliable marrow quantification is difficult to achieve, and manual segmentation is impractical in clinical workflows. Most automated tools focus on solid tumors and lack clinical validation for skeletal FLT-PET/CT. This study evaluates deep learning whole-body segmentation and cortical–trabecular marrow quantification on FLT-PET/CT in AML with HCT.

**Results:**

Twenty refractory AML patients undergoing total marrow and lymphoid irradiation (TMLI) and transplantation were analyzed. From 134 predefined regions, five representative ROIs (spleen, liver, T6, L1, L3) validated agreement with manual segmentation. Automated and manual count measurements showed strong agreement, with a high correlation (r > 0.98, p < 0.0001). Consistent hotspot detection by both methods supports the AI tool’s accuracy and clinical applicability. Small liver/spleen differences and larger positive vertebral trabecular biases were observed. AI cut processing time by ~ 95%, markedly improving efficiency.

**Conclusion:**

This study provides a technical validation of an AI-driven multi-organ segmentation platform for FLT-PET/CT in AML and HCT, including separate cortical bone and trabecular marrow compartments. The automated approach demonstrated high agreement, excellent reproducibility, and substantial efficiency gains in skeletal marrow and organ quantification. These findings establish a scalable framework for future studies that will correlate FLT-based bone marrow metrics with clinical response and transplant outcomes.

**Trial registration:**

ClinicalTrials.gov NCT03422731. Registered 6 February 2018, https//www.cancer.gov/research/participate/clinicaltrialssearch/v?id=NCI201701778

## Introduction

[18F] 3’-deoxy-3’-fluorothymidine (F-18 FLT) is a PET tracer of cellular proliferation that is trapped after phosphorylation by thymidine kinase-1 in cycling cells (1, 2). It accumulates in hematopoietic bone marrow, spleen, and sites of leukemia involvement, and has been applied to detect acute myeloid leukemia (AML), assess treatment response, and monitor engraftment after hematopoietic cell transplantation (HCT) (3). However, reliable quantification of FLT uptake in bone marrow is challenging because trabecular spaces are small, anatomically complex, and show low contrast on CT, while manual delineation is time-consuming and impractical for routine clinical use or multi-timepoint analysis.

Deep learning based multi organ segmentation has improved performance over classical thresholding, region growing, and atlas based methods, and is increasingly used in diagnostic and radiotherapy workflows (4–9). Existing AI tools for bone and marrow imaging have focused mainly on CT based bone mineral density, bone marrow lesions on MRI, or CT body composition analysis rather than tracer activity measurement, and they generally do not distinguish cortical from trabecular bone. This limitation is important because biologically meaningful marrow proliferation occurs primarily in trabecular compartments.

In this study, we evaluated a deep learning based AI platform for automated multi-organ CT segmentation and cortical–trabecular marrow separation, coupled to FLT-PET quantification, in patients with AML undergoing HCT. We compared automated and expert manual segmentations for representative organs and trabecular vertebral regions and characterized skeletal, muscle, fat, and organ FLT uptake using the automated workflow. To our knowledge, this is the first clinical technical validation of whole body cortical and trabecular marrow segmentation for FLT-PET/CT in AML and HCT.

## Materials and methods

### Patient Selection

Twenty patients with refractory acute myeloid leukemia (AML) scheduled to undergo total marrow and lymphoid irradiation (TMLI) combined with chemotherapy, followed by stem cell transplantation, were included in this analysis at the pre-treatment time point. All patients were enrolled on an ongoing clinical trial of FLT-PET/CT imaging (NCT03422731). Of these, 12 patients underwent whole-body FLT-PET/CT from head to toe, and 8 underwent upper-body imaging from neck to femur. The study was approved by the City of Hope Institutional Review Board (protocol 17222) and all participants provided written informed consent in accordance with institutional and regulatory guidelines. The mean age was 54.8 ± 13.6 years (range 20–75 years), mean height was 171 ± 8 cm, and mean weight was 80.0 ± 14.0 kg.

#### [^18^F] FLT-FLT PET/CT Imaging Protocols

All imaging was obtained using an integrated PET/CT scanner (Optima 560, GE Medical Systems, USA). [^18^F] FLT (2.06 ± 0.82 MBq/kg) in 2–5 mL of normal saline was injected intravenously. PET/CT (Optima 560, GE Medical Systems, USA) imaging was performed one hour after the [^18^F] FLT injection, covering the region from the vertex to the upper thigh. A standardized helical CT protocol (140 kVp, standard kernel) was used. The upper-body scan was acquired at ~ 270 mA with a voxel size of 1.38 × 1.38 × 3.27 mm^3^, while the lower-body scan used ~ 200 mA, a 0.875 pitch, and a voxel size of 0.98 × 0.98 × 3.75 mm^3^. PET data were then acquired immediately for 1 min per bed position. PET images comprised approximately 600 ± 40 slices for the whole body varying according to patient height, and voxel sizes of 3.65 × 3.65 × 3.27 mm^3^.

## Manual and automated segmentation

**Figure 1:**
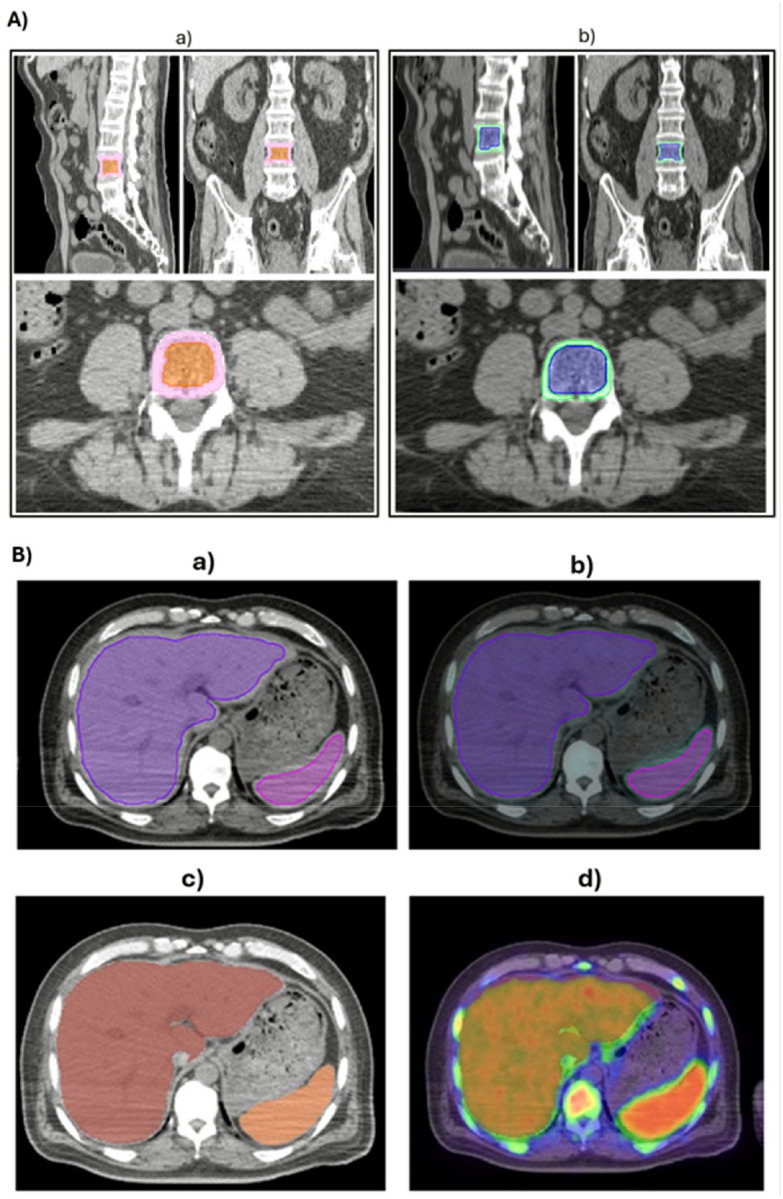
Overall segmentation of vertebral structures and Thoracoabdominal organs. **A)** Vertebral cortical and trabecular bone segmentation shown in axial, sagittal, and coronal views, including (a) automated segmentation and (b) manual segmentation. **B)**Liver and spleen segmentation on CT images, including (a) manual segmentation, (b) PET-CT map in the Velocity workspace, (c) automated segmentation, and (d) PET-CT map in the DAFS software.

For the detailed manual versus automated comparison, five representative ROIs (liver, spleen, T6, L1, and L3 trabecular compartments) were manually delineated in a subset of 8 patients, selected at random from the patient cohort. Manual segmentation and site-wise quantification were performed using Velocity (Fig. 1.A.b and Fig. 1.B.a), a routine image processing platform within the radiation oncology workflow (Varian Medical Systems, Palo Alto, CA), on CT-scan images. Manual segmentation was carried out by three observers, including two medical imaging experts with varying levels of segmentation experience and one radiologist. The operators performing manual segmentation did not require additional training to use the Velocity software, as it is a platform routinely employed in our research workflow.

Automated segmentation was performed using the data analysis facilitation suite (DAFS; Voronoi Health, Canada), (Fig. 1.A.a and Fig. 1.B.c). For DAFS input, no specific data preparation was required; CT and PET images (DICOM format) were simply imported into the software as a common folder. The segmentation covers muscles, fat compartments, major organs, bones (cortical and trabecular), vessels, glands, and pathological regions, with datasets processed with and without upper limbs for standardized quantification. The resulting masks provided volumetric and compositional data across multiple tissue and organ systems, enabling detailed morphometric and metabolic analyses. To this end, each CT annotation and segmentation was inspected via a sagittal, coronal, and axial view of each scan using the quick check quality option; mis-annotations were corrected using the CAST (CT Annotation and Segmentation Tool) feature from DAFS. Using DAFS, a total of 134 anatomical regions can be defined, categorized into skeletal muscle (n = 23), adipose tissue (n = 28), bone (n = 49), and organs, soft tissues, and glands (n = 34).

**Figure 2:**
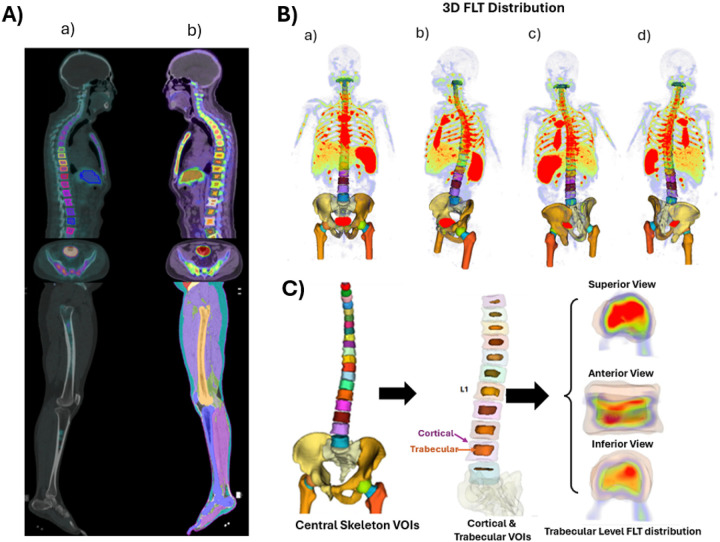
**(A) Segmentation of thoracic and lumbar vertebrae and the liver. (a)** Manual segmentation performed in Velocity and **(b)**automated segmentation generated by DAFS in whole-body FLT PET/CT images. **(B) Macroscopic FLT distribution (3D):** Automated upper-body 3D renderings illustrating the spatial distribution of FLT uptake across the skeleton, with multi-view visualization of the spine, hips, and proximal femora: (a) anterior, (b) anterior oblique, (c) posterior, and (d) posterior oblique. **(C) Compartment-specific visualization highlighting FLT activity within trabecular bone marrow, enabling detailed assessment of marrow proliferation independent of cortical bone signal:** (a) central skeletal segmentation; (b) cortical (purple arrow) and trabecular (orange arrow) compartments of the thoracic and lumbar vertebrae, including a 3D rendering of the trabecular compartment; (c) microscopic-level FLT-PET mapping of the trabecular region of the L1 vertebra (superior view); (d) anterior view; and (e) inferior view.

Automated upper-body 3D renderings were generated to depict macroscopic FLT distribution across the trunk (Fig. 2. B, a–d). Cortical bone and trabecular marrow were automatically separated to enable compartment-specific analysis. Trabecular-focused visualization highlighted FLT activity within marrow space, including detailed cortical-trabecular delineation in thoracic and lumbar vertebrae and microscopic FLT mapping of the L1 trabecular region in superior, anterior, and inferior views (Fig. 2. C).

To ensure an unbiased comparison between manual and automated segmentation and to avoid variables that could introduce error, Bq/mL values were used. Body-weight–normalized Standardized Uptake Value (SUV) measurements from all

ROIs are reported as complementary results. Body-weight–normalized SUV (SUVbw) was calculated using Eq. 1:

(Eq. 1) $$\text{SUV}=\frac{\text{Activity}(\text{Bq}/\text{mL})\times \text{Body}\;\text{weight}(\text{kg})\times 1000(\text{g}/\text{kg})}{\text{Injected}\;\text{Dose}(Bq)*2^{-\left(\frac{\text{Aquisition}\;\text{Time}-\text{StartTime}}{\text{Half}\;\text{Life}}\right)}}$$

In this formulation, the tissue activity concentration (Bq/mL) was obtained from the PET images, and the injected dose (Bq) was decay-corrected to the time of acquisition using the physical half-life of the radiotracer. Body weight (kg) was incorporated into the numerator to normalize uptake across subjects. All SUVbw calculations were performed using Microsoft Excel (Microsoft Corp., USA) formula to ensure consistent quantification across subjects.

### Statistical Analysis

Pearson’s (r ) and Spearman’s (r ) correlation coefficients, accounting for data normality, along with Bland-Altman (B&A) analysis, were used to compare Bq/ml values across all ROIs between the two methods. Inter-operator reliability was evaluated using the intraclass correlation coefficient (ICC) between three independent operators (medical imaging experts and a radiologist) performing manual segmentation. Normality of cortical and trabecular SUV measurements was assessed using the Shapiro–Wilk test. As normality assumptions were not met, group differences were evaluated with the nonparametric Mann–Whitney U test (two-tailed). a two-sided *p* value < 0.05 was considered statistically significant.

## AI-Assisted Editing

AI-assisted tools (ChatGPT, OpenAI) were used only for language editing and improving clarity in the manuscript text. All scientific content, analyses, and interpretations were performed and verified by the authors.

## Results

**Figure 3:**
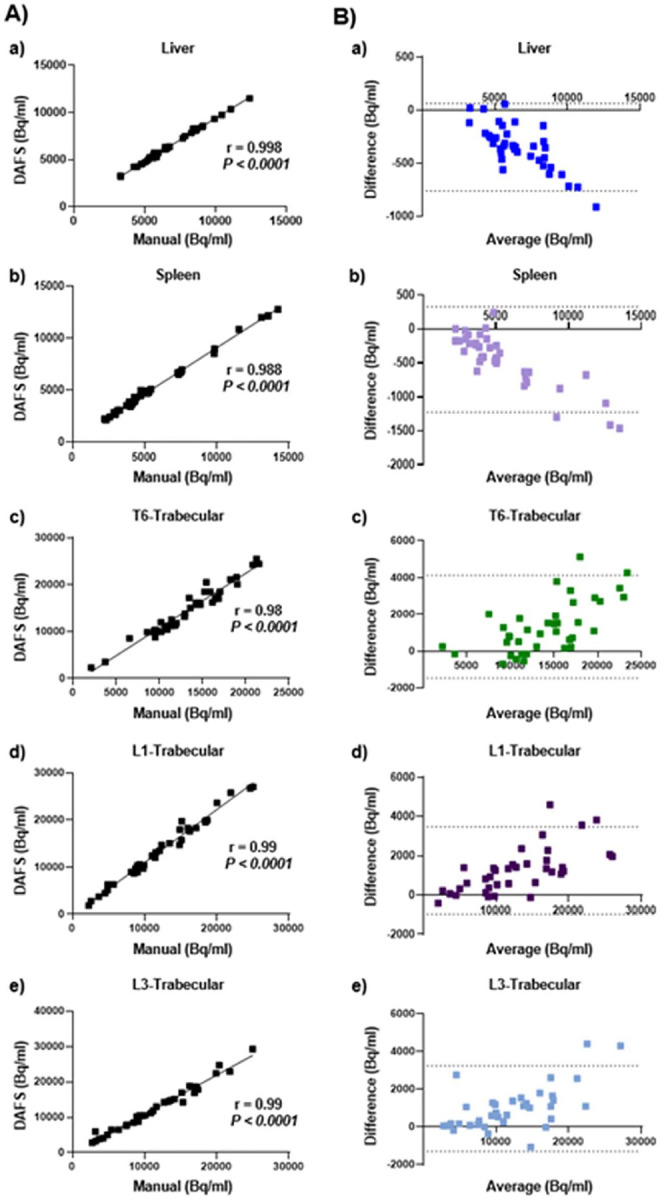
Correlation (Pearson’s *r*_*p*_ and Spearman’s *r*_*s*_) and Bland-Altman (B&A) analyses of mean Bq/mL values comparing automated and manual segmentations. **(A)** Correlation plots for manual segmentation from 8 patients in (a) liver, (b) spleen, (c) T6 trabecular bone, (d) L1 trabecular bone, and (e) L3 trabecular bone regions versus automated segmentation. **(B)** B&A analysis, showing the difference between the two segmentation methods (Manual − Automated), plotted against the mean values (Manual+Automated)/2) in (a) liver, (b) spleen, (c) T6 trabecular bone, (d) L1 trabecular bone, and (e) L3 trabecular. The mean difference (solid line) and limits of agreement (dotted line) are shown. B&A, Bland & Altman; r_p_, Pearson’s correlation coefficient, r_s,_ Spearman’s correlation coefficient.

The agreement between automated and manual segmentations was assessed using Pearson’s (rp) and Spearman’s (rs) correlation coefficients to evaluate their linear relationship (Fig. 3A, a–e). Across the five representative ROIs (liver, spleen, and trabecular compartments of T6, L1, and L3), Pearson correlation coefficients (r ) ranged from 0.988 to 0.998 (p < 0.0001 for all comparisons). Spearman correlation for the spleen was 0.983, indicating strong monotonic agreement. Moreover, the agreement between manual and automated was evaluated by employing B&A analysis (Fig. 3B, a–e), where the difference between the two methods (Manual-Automated) was plotted against the mean values of them (Manual+Automated)/2). The mean differences are − 354.5, −448.9, 1332.0 and 961.2 Bq/mL for a) liver, b) spleen, c) T6 trabecular, d) L1 trabecular and e) L3 trabecular, respectively.

**Figure 4:**
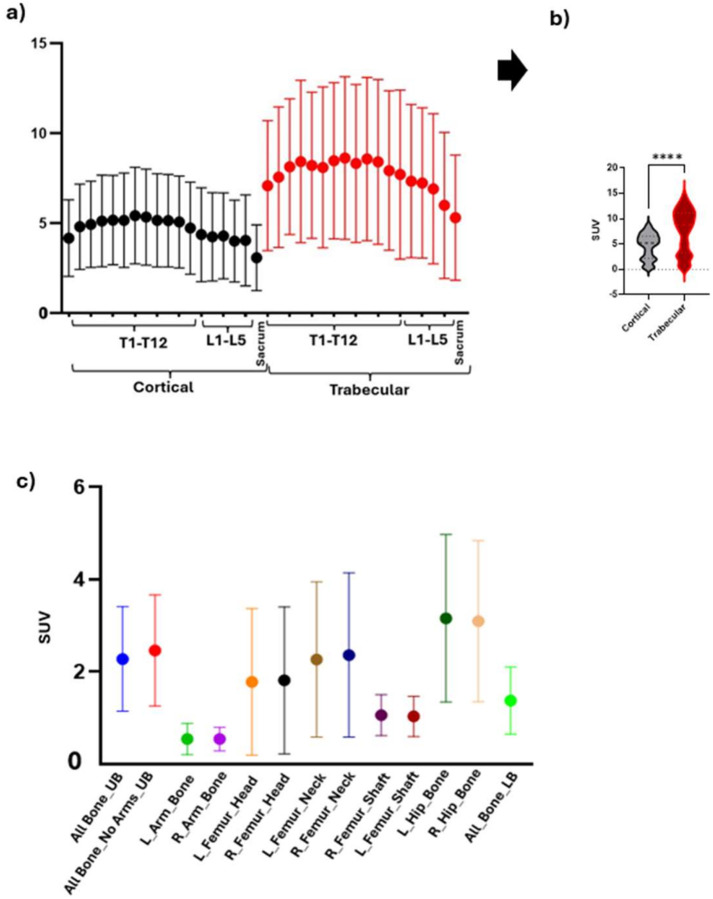
Skeletal SUVs from FLT-PET/CT. **(a)** Spinal cortical, and trabecular compartments across thoracic levels T1–T12, lumbar levels L1–L5, and the sacrum. **(b)** Violin plots showing SUV distributions across cortical bone and trabecular marrow compartments. The trabecular marrow shows significantly higher values than cortical bone.**** (p < 0.0001), **(c)** Skeletal bone groups. UB=Upper body; Lower body=LB.

**Figure 5:**
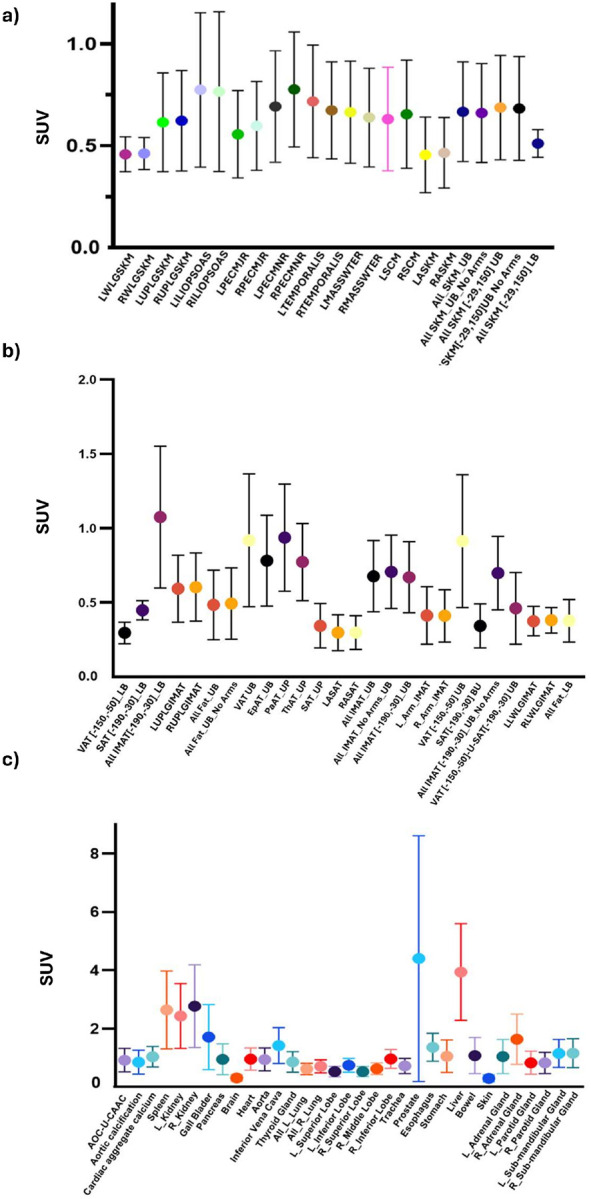
SUVs from FLT-PET/CT across (a) skeletal muscle, (b) adipose tissue, and (c) other organs UB=Upper body; Lower body=LB, All SKM = all skeletal muscles; All SKM[−29,150] = all skeletal muscles within HU −29 to 150; No Arms = excluding upper limbs; LPECMJR/RPECMJR = left/right pectoralis major; LPECMNR/RPECMNR = left/right pectoralis minor; LTEMPORALIS/RTEMPORALIS = left/right temporalis; LMASSETER/RMASSETER = left/right masseter; LSCM/RSCM = left/right sternocleidomastoid; LILIOPSOAS/RILIOPSOAS = left/right iliopsoas; LUPLGSKM/RUPLGSKM = left/right upper limb muscles; LLWLGSKM/RLWLGSKM = left/right lower limb muscles; LASKM/RASKM = left/right abdominal muscles; AOC-U-CAAC= enables AOC-CAAC-based agatston score; VAT = visceral adipose tissue; VAT[−150,−50] = visceral fat within HU −150 to −50; EPAT = epicardial/pericardial fat; PAAT = periaortic fat; THAT = thoracic fat; SAT = subcutaneous fat; SAT[−190,−30] = subcutaneous fat within HU −190 to −30; LASAT/RASAT = left/right abdominal subcutaneous fat. IMAT = intramuscular adipose tissue; All_IMAT [−190, −30] = IMAT within HU −190 to −30; LUPLGIMAT/RUPLGIMAT = left/right upper limb IMAT; LLWLGIMAT/RLWLGIMAT = left/right lower limb IMAT; LAIMAT/RAIMAT = left/right abdominal IMAT.

SUV values for all organ quantified ROIs are illustrated in Figs. 4 and 5 and **Tables S1- S3**. Figure 4a demonstrates distinct trabecular SUV values across the thoracic, lumbar, and sacral spine compared with cortical. The trabecular marrow showed significantly higher values compared with cortical bone (p < 0.0001), (Fig. 4b). Figure 4c presents skeletal bone SUVs. In our cohort, muscle and fat ROIs showed uniformly low FLT uptake, as expected for non-proliferative tissues (Fig. 5a, b, **Table S2**), confirming that the AI workflow performs robustly across diverse tissue classes and functions as a true multi-organ tool rather than a marrow-only application. Notably, elevated SUVs in lower-body regions defined by − 150 to − 50 HU and in visceral adipose tissue (VAT) were present in approximately 50% of the cohort and are therefore detailed in **Tables S2** rather than illustrated in the figure. Among organ SUVs (Fig. 5c), the bladder showed the highest uptake (65.450 ± 43.39), whereas the skin demonstrated the lowest values (0.299 ± 0.12). Owing to its disproportionately high uptake, the bladder was excluded from the organ plot in Fig. 5c to avoid scaling distortion; corresponding values are provided in **Tables S1–S3**.

Manual segmentation required 25 ± 4 minutes on average to delineate five organs (≈ 5 minutes per organ). In the worst-case scenario, manual delineation extended to 96.03 ± 22 minutes, or approximately 19.2 minutes per organ. In contrast, the automated method completed segmentation of 134 ROIs in 115–120 minutes (scan number: 306.42 ± 18.83), equivalent to about 0.83 minutes per ROI. When normalized per region, automation reduced processing time by approximately 84% compared with the average manual workflow, and by up to 96% compared with the worst-case manual scenario. These findings highlight the substantial efficiency gains achieved through automation, especially given that delineation time increases nearly linearly with the number of slices.

Moreover, overall inter-observer reliability was excellent, with an averaged-measures ICC = 0.97 (95% CI 0.95–0.98, n = 40 ROIs). These values showed excellent agreement, confirming the reproducibility and minimal operator dependence of the segmentation, establishing a reliable ground truth for validating the automated method. Moreover, manual inspection was performed to verify whether the software correctly segmented the targeted organs. All vertebral cancellous bones were correctly identified except for T9 and L5, which required manual correction due to aortic calcifications at T9 and increased angulation for L5, respectively.

## Discussion

In this study, we evaluated manual versus automated CT-based segmentation methods to quantify activity counts from FLT-PET/CT images in patients with AML undergoing HCT. The AI-driven multi-organ platform showed excellent agreement with expert manual segmentation for both large soft-tissue organs and small trabecular vertebral ROIs, with Pearson correlation coefficients above 0.98 and narrow B&A limits of agreement. Manual inter-observer reliability was also excellent, providing a robust ground truth for validating the automated method. These findings support the feasibility of applying deep learning-based tools for skeletal FLT-PET/CT analysis in hematologic malignancies, consistent with prior advances in multi-organ segmentation and radiotherapy imaging applications.(4–8, 10–12)

In this study, manual segmentation showed excellent correlation with the automated method across both small (T6, L1, L3 trabecular) and large organs (liver and spleen; r = 0.99–1) (Fig. 3A, a–e). B&A analysis demonstrated good agreement between methods (Fig. 3B, a–e), with most measurements falling within the limits of agreement and minimal overall bias. Agreement was strongest for large soft-tissue organs, whereas greater variability was observed in trabecular bone regions, suggesting that automated segmentation may be more challenged by fine bone structures despite overall consistency.

In trabecular spinal regions (T6, L1, L3) showed a slight positive bias, likely reflecting sharper bone boundary definition on CT. Cortical thickness appeared greater in the automated segmentation than in the manual segmentation (Fig. 1A), suggesting cortical overestimation by the automated method, particularly at T6 (1332.0 Bq/mL) and L1/L3 (961.2 Bq/mL). Tighter automated contours yield smaller ROIs and higher apparent FLT uptake, underscoring the sensitivity of PET quantification to segmentation precision.

The biological separation of cortical bone and trabecular marrow is essential for accurate interpretation of FLT uptake. Trabecular bone contains hematopoietically active marrow with high cellular proliferation, whereas cortical bone is largely mineralized and minimally proliferative; accordingly, FLT signal-retained after phosphorylation by thymidine kinase-1 during the S-phase-primarily reflects proliferating hematopoietic cells.(1–3) Consistent with this mechanism, trabecular SUV measurements provide a more specific representation of marrow proliferative activity than cortical or composite vertebral values, with higher uptake demonstrated in Fig. 4a, underscoring the clinical relevance of compartment-specific analysis in refractory acute myeloid leukemia, leukemia-niche characterization, and radiation-targeting strategies. (13) Given the marked radiosensitivity of bone marrow and its role as a dose-limiting organ, accurate assessment of active red marrow is critical for transplantation and dose- toxicity evaluation. (14). Prior FLT-PET studies, including McGuire et al., have shown dose-dependent suppression of marrow proliferation with vertebral uptake correlating with delivered radiation dose, supporting FLT-PET as a sensitive biomarker of hematopoietic activity.(15) Precise trabecular-specific quantification therefore enables improved evaluation of marrow response, recovery, and clinical outcomes. Three-dimensional automated renderings further illustrate the macroscopic spatial distribution of FLT uptake across the upper-body skeleton, consistently visualizing the spine, hips, and proximal femora from multiple viewing angles (Fig. 2B, a–d). Global active marrow mapping enables skeleton-wide assessment of hematopoiesis, heterogeneity, disease niches, and post-transplant regeneration beyond localized biopsy. Trabecular-specific segmentation isolates FLT activity from cortical signal (Fig. 2C), improving biologically precise assessment of microscopic proliferation and reducing structural, partial-volume, and mineral attenuation confounding relevant to therapy and transplantation.(3, 7–9, 16)

In manual segmentation for multi organs, organ boundaries, such as the liver, are often drawn slightly within the true anatomical edge to reduce partial-volume effects. However, due to limited soft-tissue contrast in CT, operators place the contour several pixels within the actual margin (often exceeding the ideal ~ 3-pixel offset). As a result, manual segmentations can fall noticeably within the true organ boundary; consequently, manual segmentation tends to overestimate mean values in soft-tissue regions such as the liver and spleen, as derived from CT images. In contrast, DAFS identifies the external anatomical boundary algorithmically. Ideally, once the exact outer border is defined, the software should then apply a standardized inward offset (e.g., 3 pixels) to minimize partial volume effects while avoiding the operator-dependent inward bias seen in manual segmentation (Fig. 1.B.c). No study confirms outer-voxel selection in automated segmentation, but PET boundary voxels commonly show mixed tissue and underestimated activity. (17) Manual contouring is highly variable, while automated methods consistently capture complete organ boundaries (4), which may contribute to the lower liver and spleen uptake observed with automated segmentation.

SUV values across regions are summarized in Figs. 4–5 and **Tables S1–S3**. The platform also segmented muscle, adipose tissue, and major organs, with uniformly low FLT uptake in muscle and fat confirming robust multi-organ performance. The prostate exhibited the highest SUV (4.403 ± 4.23) among the organ category, with the substantial variation potentially attributable to size-related differences.

Organ segmentation is essential for imaging and therapy but remains limited by traditional thresholding, (18), graph-cut (19), region-growing (20), and atlas-based methods (21), which show variability, computational burden, and poor performance in irregular or low-contrast anatomy. (4) Deep learning- particularly transformer models such as Swin UNETR-has improved accuracy, robustness, and generalizability in whole-body PET/CT segmentation. However, standardized evaluation of clinical efficiency, especially in non-solid tumors, is lacking. Current artificial intelligence applications in bone marrow imaging largely focus on magnetic resonance–based density or lesion assessment, (4, 7, 8, 12, 22). and DAFS has primarily been used for computed tomography body composition rather than FLT-PET/CT (23).

Most CT segmentation tools cover limited organs, with some reaching ~ 120 structures(10), whereas the proposed software segments 134–152. Because HU-based algorithms cannot separate cortical from trabecular bone, (11) essential for FLT marrow assessment (10), DAFS offers broader coverage, reproducibility, usability, and compartment-specific distinction.

Finally, automated segmentation markedly improved efficiency compared with manual delineation. This highlights its superior scalability for volumetric datasets, where manual delineation time increases almost linearly with the number of slices.

Beyond speed, reducing user interaction minimizes operator fatigue and variability, and common challenges in manual workflows. Collectively, these findings underscore the practicality of automated segmentation for large-scale or time-sensitive imaging studies, where accuracy and consistency throughput are critical for clinical and research applications.

This work still has several limitations. Firstly, our validation was evaluated using data from a single institution and a single scanner. As a result, more studies using data from several other institutions are needed to demonstrate the generalizability of the results. The ROI mismatch between manual and the automatic software system for T9 and L5 trabecular and cortical segmentation is also a limitation in this study. Moreover, because the whole-body scan contains overlapping slices between the upper- and lower-body acquisitions, redundant slices can be removed in future studies, leaving only the selected non-overlapping slices for import into the DAFS software for quantification.

Despite these limitations, our findings indicate that AI-based multi-organ segmentation with explicit cortical and trabecular marrow compartmentalization can provide accurate, reproducible, and efficient FLT-PET/CT quantification in patients with AML undergoing HCT. By extending prior multi-organ and bone marrow segmentation work to functional FLT imaging, this study establishes a technical foundation for future investigations that will link regional marrow activity to clinical and biological endpoints, including biopsy targeting, treatment response assessment, and risk stratification in leukemia and transplant care.

## Conclusion

In this study, we implemented and validated a fully automated three-dimensional segmentation and quantification workflow using the DAFS platform to measure regional FLT activity (Bq/mL) on PET/CT in patients with AML undergoing HCT. To our knowledge, this is the first technical multi-organ validation of automated cortical bone and trabecular marrow segmentation for FLT-PET/CT in this setting. The AI-based approach showed excellent agreement with expert manual segmentation while enabling rapid, reproducible, and largely operator-independent assessment of skeletal marrow and organ activity. Among currently available multi-organ segmentation platforms, DAFS is notable for its explicit separation of cortical bone and trabecular marrow, a feature that is essential for biologically specific evaluation of active bone marrow. By accelerating PET analysis and standardizing marrow and organ quantification, this validated workflow provides a scalable foundation for future studies that will link FLT-based marrow metrics to clinical and biological endpoints and may ultimately support noninvasive assessment of functional bone marrow in leukemia and transplant care.

## Supplementary Material

Supplementary Files

This is a list of supplementary files associated with this preprint. Click to download.

- Summlementary1.Malekzadehetal.pdf

## Data Availability

The data that support the findings of this study are available from the corresponding author upon reasonable request.

## Abbreviations

FLT-PET: [^18^F] 3’-deoxy-3’-fluorothymidine positron emission tomography
AML: acute myeloid leukemia
HCT: hematopoietic stem cell transplant
TMLI: total marrow and lymphoid irradiation
ROIs: regions of interest
DAFS: data analysis facilitation suite
ICC: intraclass correlation coefficient
TK1: thymidine kinase-1
BM: bone marrow
CAST: CT annotation and segmentation tool
SUV: standardized uptake value
SUVbw: body-weight–normalized SUV
UB: upper body
LB: lower body
All SKM: all skeletal muscles
All SKM [−29,150]: all skeletal muscles within HU −29 to 150
LPECMJR/RPECMJR: left/right pectoralis major
LPECMNR/RPECMNR: left/right pectoralis minor
LTEMPORALIS/RTEMPORALIS: left/right temporalis
LMASSETER/RMASSETER: left/right masseter
LSCM/RSCM: left/right sternocleidomastoid
LILIOPSOAS/RILIOPSOAS: left/right iliopsoas
LUPLGSKM/RUPLGSKM: left/right upper limb muscles
LLWLGSKM/RLWLGSKM: left/right lower limb muscles
LASKM/RASKM: left/right abdominal muscles
AOC: aortic calcification
CAAC: cardiac aggregate calcium
VAT: visceral adipose tissue
VAT [−150, −50]: visceral fat within HU −150 to −50
EPAT: epicardial/pericardial fat
PAAT: periaortic fat
THAT: thoracic fat
SAT: subcutaneous fat
SAT [−190, −30]: subcutaneous fat within HU −190 to −30
LASAT/RASAT: left/right abdominal subcutaneous fat
IMAT: intramuscular adipose tissue
All IMAT [−190, −30]: IMAT within HU −190 to −30
LUPLGIMAT/RUPLGIMAT: left/right upper limb intramuscular adipose tissue
LLWLGIMAT/RLWLGIMAT: left/right lower limb intramuscular adipose tissue
LAIMAT/RAIMAT: left/right abdominal intramuscular adipose tissue
